# Genome-Wide Association Studies in Japanese Quails of the F_2_ Resource Population Elucidate Molecular Markers and Candidate Genes for Body Weight Parameters

**DOI:** 10.3390/ijms26178243

**Published:** 2025-08-25

**Authors:** Natalia A. Volkova, Michael N. Romanov, Nadezhda Yu. German, Polina V. Larionova, Anastasia N. Vetokh, Ludmila A. Volkova, Alexander A. Sermyagin, Alexey V. Shakhin, Darren K. Griffin, Johann Sölkner, John McEwan, Rudiger Brauning, Natalia A. Zinovieva

**Affiliations:** 1 L. K. Ernst Federal Research Center for Animal Husbandry, Dubrovitsy, Podolsk 142132, Russia; natavolkova@inbox.ru (N.A.V.); ngerman9@gmail.com (N.Y.G.); volpolina@mail.ru (P.V.L.); anastezuya@mail.ru (A.N.V.); ludavolkova@inbox.ru (L.A.V.); alexshahin@mail.ru (A.V.S.); 2 School of Natural Sciences, University of Kent, Canterbury CT2 7NJ, UK; d.k.griffin@kent.ac.uk; 3 Animal Genomics and Bioresource Research Unit (AGB Research Unit), Faculty of Science, Kasetsart University, Chatuchak, Bangkok 10900, Thailand; 4 Russian Research Institute of Farm Animal Genetics and Breeding—Branch of the L. K. Ernst Federal Research Centre for Animal Husbandry, Pushkin, St. Petersburg 196625, Russia; alex_sermyagin85@mail.ru; 5 BOKU University, 1180 Vienna, Austria; johann.soelkner@boku.ac.at; 6 AgResearch, Invermay Agricultural Centre, Mosgiel 9053, New Zealand; john.mcewan@agresearch.co.nz (J.M.); rudiger.brauning@agresearch.co.nz (R.B.)

**Keywords:** Japanese quail (*Coturnix japonica*), molecular markers, genome-wide association study (GWAS), genotyping-by-sequencing (GBS), single nucleotide polymorphisms (SNPs), candidate genes, body weight

## Abstract

Molecular research for genetic variants underlying body weight (BW) provides crucial information for this important selected trait when developing productive poultry breeds, lines and crosses. We searched for molecular markers—single nucleotide polymorphisms (SNPs)—and candidate genes associated with this trait in 240 F_2_ resource population Japanese quails (*Coturnix japonica*). This population was produced by crossing two breeds with contrasting growth phenotypes, i.e., Japanese (with lower growth) and Texas White (with higher growth). The birds were genotyped using the genotyping-by-sequencing method followed by a genome-wide association study (GWAS). Using 74,387 SNPs, GWAS resulted in 142 significant SNPs and 42 candidate genes associated with BW at the age of 1, 14, 28, 35, 42, 49 and 56 days. Hereby, 25 SNPs simultaneously associated with BW at more than one age were established that colocalized with nine prioritized candidate genes (PCGs), including *ITM2B*, *SLC35F3*, *ADAM33*, *UNC79*, *LEPR*, *RPP14*, *MVK*, *ASTN2*, and *ZBTB16*. Twelve PCGs were identified in the regions of two or more significant SNPs, including *MARCHF6*, *EGFR*, *ADGRL3*, *ADAM33*, *NPC2*, *LTBP2*, *ZC2HC1C*, *SATB2*, *ASTN2*, *ZBTB16*, *ADAR*, and *LGR6*. These SNPs and PCGs can serve as molecular genetic markers for the genomic selection of quails with desirable BW phenotypes to enhance growth rates and meat productivity.

## 1. Introduction

Research on the molecular basis for the formation of physiological traits and genetic variants in livestock [1,2], including poultry breeds [3,4,5,6,7], is an important prerequisite for the implementation of promising performance and adaptation phenotypes [8,9,10,11]. It has practical use in genomic selection, forecasting and assessment of productive and reproductive potential [12,13,14,15,16]. The development and competitiveness of industrial poultry farming largely depends on the creation and use of high productive breeds and crosses characterized by improved egg and meat qualities [17,18,19,20,21,22]. This is impracticable to implement without effective selection work aimed at searching for and identifying valuable molecular markers and genotypes [23,24,25,26,27]. Such work makes use of modern high-throughput and whole-genome methods and approaches [28,29,30,31,32] based on the study of physiological and molecular genetic mechanisms of the formation and manifestation of selectively significant traits [33,34].

Body weight (BW) is one of the main traits taken into account in poultry breeding and selection. This is particularly the case when developing commercial crosses and lines for the production of both meat and eggs [35,36,37,38]. BW indicator and the dynamics of its change during ontogenesis serve as an important criterion for monitoring the development, assessment and prediction of the productive qualities of poultry [39,40,41,42]. A number of studies have established correlations between BW and linear measurements [39,40,41,43]. It has been shown that higher BW in the early ontogenesis period of birds positively correlates with rapid growth, higher BW and good meat qualities of birds at a later age [42,44,45]. Smaldone et al. [46] reported the results of studies on the effectiveness of using two commercially available types of electrical equipment for stunning broilers at slaughter, taking into account their BW. An inverse relationship was found between broiler BW and the number of carcass lesions (i.e., hemorrhages and dark meat).

BW indicator is also important in egg poultry farming where BW of laying hens correlates with their egg performance and quality [45,47]. In particular, Raziq et al. [47] showed that laying hens with a higher BW at the beginning of egg laying, compared to their peers with lower or average BW, were characterized by a higher BW at maturity, an earlier maturation period and a higher egg weight. Herewith, hens with a lower BW at the beginning of egg laying had a higher egg production. A negative correlation between BW of laying hens and egg production was also confirmed for Japanese quails (*Coturnix japonica*; CJA) [48]. Notably, a positive effect of selection for higher BW of quail females at an early age on the egg weight indicator was noted [45].

The BW indicator is influenced by a number of factors. The growth potential of poultry is determined primarily by its genotype. Numerous studies have shown the genetic determination of this trait in different poultry species, in particular, chickens [36,49,50], quails [43,51,52,53], ducks [54,55,56,57,58] and turkeys [17,59]. The implementation of the genetic growth potential largely depends on feeding [60,61,62] and housing conditions [63].

To date, extensive information has been accumulated on genetic markers associated with BW and growth rate in different types of poultry, especially when using whole-genome genotyping and sequencing [64,65,66,67,68,69]. Herewith, a significant proportion of such studies were performed on chickens. This is facilitated by the availability of commercial genome-wide DNA arrays for molecular genetic studies available for this poultry species [31,70,71,72,73,74]. There are no such commercial genomic analysis systems for quail, however, which relatively limits the research to find loci and candidate genes that determine the degree of manifestation of economically important phenotypes. In this regard, research in this area of quail molecular genetics is relevant and in demand.

In our earlier studies using the genotyping-by-sequencing (GBS) technique, we analyzed potential genes and selection signatures in eight quail breeds [75]. We identified SNPs and prioritized candidate genes (PCGs) associated with abdominal fat deposition, growth and meat productivity in F_2_ quails from a resource population obtained using two breeds, i.e., Japanese (of lower growth rate) and Texas White (of higher growth rate) [52,76]. The aim of the current study was to extend these previous works to focus in more detail on the search for SNPs and identification of candidate genes associated with BW at different age periods, specifically at 1 (BW1), 7 (BW7), 14 (BW14), 21 (BW21), 28 (BW28), 35 (BW35), 42 (BW42), 49 (BW49) and 56 (BW56) days of age. In line with this aim, a genome-wide association study (GWAS) for BW indices was performed in F_2_ quails of different ages from the same cross of Japanese (with average BW) and Texas White (with higher growth rate) breeds based on GBS genotyping data.

## 2. Results

### 2.1. F_2_ Resource Population Phenotypic Data and Population Stratification

Descriptive metrics for the age dynamics of BW in F_2_ quails of the resource population in comparison with the original parent breeds are presented in Table 1. At all studied ages, F_2_ quails exceeded Japanese quails in this indicator by 4–26% and were inferior to Texas White quails by 6–25%, i.e., tended to demonstrate intermediate values. The maximum differences in the studied BW traits of the resource population quails and the original parent breeds were noted in a later age period. It should be noted that there was a higher variability of the established BW values in the resource population quails compared to Japanese and Texas White quails at the age of 7 and 14 days.

Table 2 shows the results of computing the heritability coefficients and variation components. The highest heritability was noted in quails at the age of one day (0.565) with a smooth decrease in genetic variability for BW14 (0.312). Over the investigation period, starting from 21 to 56 days of age, minor fluctuations in the heritability coefficients for BW of individuals were observed in dynamics (from 0.277 to 0.293) with a tendency for its smooth decline as the birds grow. Most likely, this may be explained by the individual age-related characteristics of the bird’s body with an increase in its BW, a change in physiological status (sexual maturity) and, accordingly, a reaction to environmental factors.

Genetic correlations between different age groups of quails in terms of BW indicators are presented in Table 3.

Genetic correlation between BW1 and the indices in subsequent age periods had low correlation values, which may indicate an independent character of the BW1 trait formation caused by prenatal development (e.g., egg size/weight, maternal age, etc.). At later ages, regular changes in the correlation dependence were observed in the dynamics. With increasing age of the birds, between adjacent indices within the compared age group, the genetic correlation lowered, thereby demonstrating the complex nature of inheritance of the BW index. It can be noted that the tightness of the genetic correlations for BW28 to BW56 was the greatest (0.492–0.751), which in turn indicates the potential for selection of individuals for further breeding according to this index.

**Figure 1:**
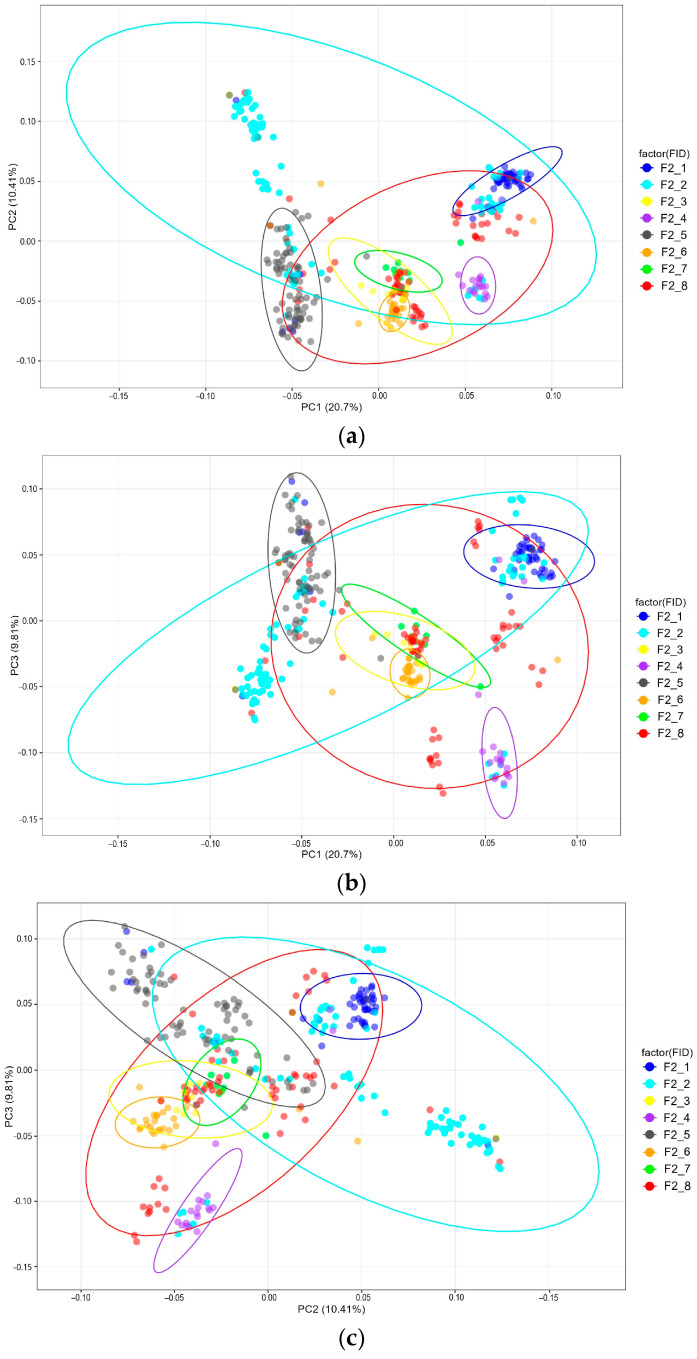
Principal component analysis (PCA) for the F_2_ quail resource population structure: (**a**) PCA performed in the plane of the first (PC1; *x*-axis) and second (PC2; *y*-axis) components; (**b**) PCA performed in the plane of PC1 (*x*-axis) and the third (PC3; *y*-axis) components. (**c**) PCA performed in the plane of PC2 (*x*-axis) and PC3 (*y*-axis). Individuals from different groups are marked with different colors.

After filtering the raw data, a total of 115,743 SNPs were generated, while 92,618 SNPs remained after quality control and were used for the subsequent analysis. To assess and characterize the SNP-based genetic structure of the F_2_ quail resource population, groups of F_2_ individuals were formed depending on their origin. Taking into account the males used to obtain F_2_ quails, eight groups were established (F2_1 to F2_8). According to the principal component analysis (PCA), the studied groups of F_2_ quails formed one a few overlapping clusters in the projections PC1–PC2, PC1–PC3 and PC2–PC3. This population stratification pattern is visually represented in Figure 1a–c).

Given the observed structure of the F_2_ resource population, a GWAS was subsequently performed using the first three PCs as covariates as outlined below.

### 2.2. Genome-Wide Association Studies

**Figure 2:**
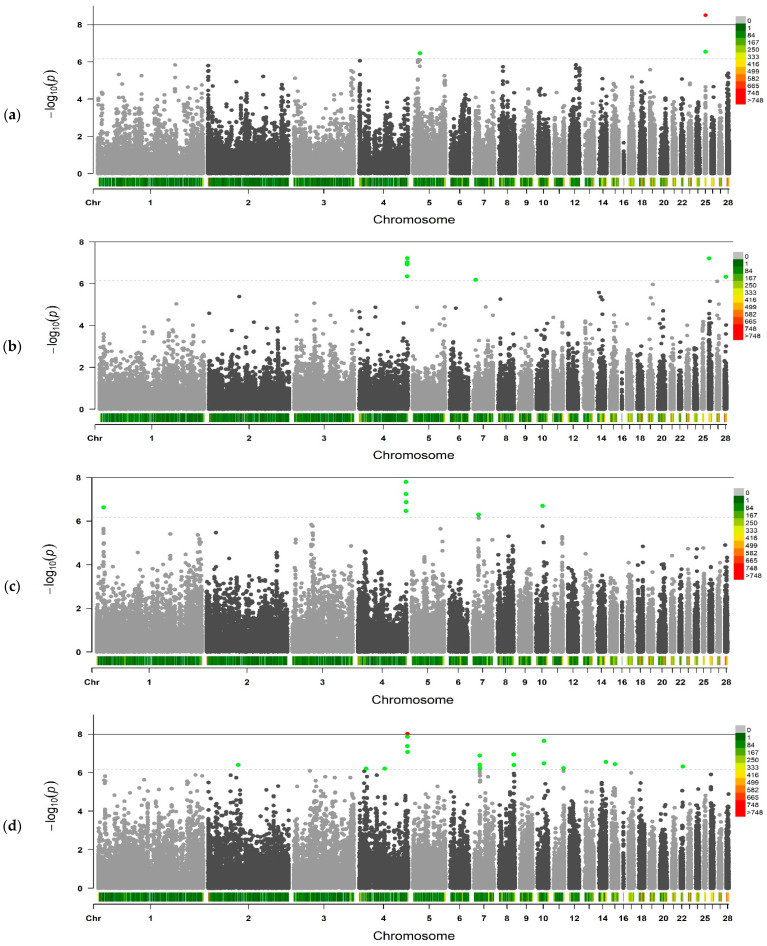

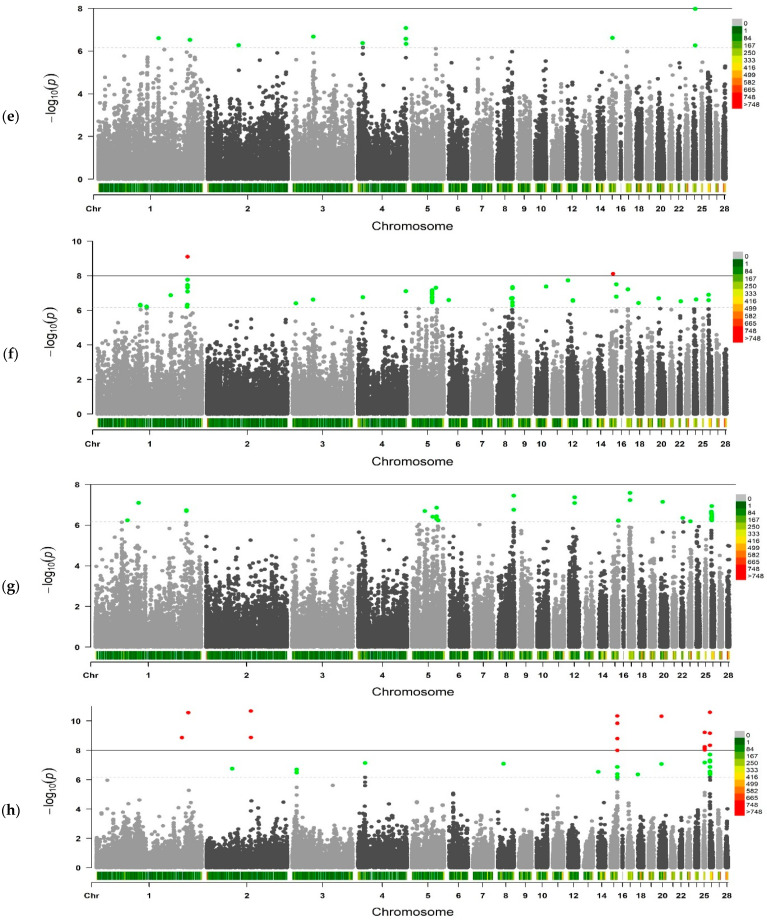
Manhattan plots based on the GWAS data for the studied body weight (BW) parameters in the F_2_ resource population of quails at the ages of 1 (**a**), 14 (**b**), 21 (**c**), 28 (**d**), 35 (**e**), 42 (**f**), 49 (**g**) and 56 (**h**) days. Manhattan plots show distribution of single nucleotide polymorphisms (SNPs) in quail chromosomes according to the significance level (−log10 (*p*)) based on the expected Bonferronni probability value of *p* < 6.72 × 10^−7^ (lower dotted line) and *p* < 1.05 × 10^−8^ (upper solid line) for the respective BW traits. SNP dots are color-coded to visualize significant values. SNPs that have significant associations with the studied traits at the level of established reliability values from *p* < 6.72 × 10^−7^ to *p* > 1.05 × 10^−8^ are highlighted in green; SNPs that are significantly associated with the traits at the level of *p* < 1.05 × 10^−8^ are highlighted in red. The diagram along the *x*-axis shows the density of established SNPs on individual chromosomes.

The experimental data on the BW indices in F_2_ quails of the genotyped resource population were used for genome-wide association studies. The GWAS results are presented in Figure 2 and Supplementary Figure S1.

The analysis performed at the level of established significance value of *p* < 6.72 × 10^−7^ revealed 142 SNPs associated with BW in the studied population quails aged from 1 to 56 days (Supplementary Table S1). These SNPs were localized on 21 of 28 chromosomes explored. Their maximum number was detected on chromosomes CJA4 (27 SNPs), CJA26 (24 SNPs) and CJA1 (20 SNPs), and the minimum one on CJA11 (1 SNP).

Data on the number of significant SNPs found and their chromosomal distribution, taking into consideration the examined BW indices of various-aged F_2_ quails in the resource population, are shown in Table 4.

The number of significant SNPs associated with BW varied depending on the age of the studied birds. The maximum number of significant SNPs associations with the studied BW trait was established at the age of 42 and 56 days (50 and 42 SNPs, respectively), and the minimum one at the age of 1 day and 14–21 days (3–8 SNPs). According to the BW BW14 indicator, no significant SNPs were identified in this study at the established threshold.

Notably, 25 SNPs associated with BW in two or more age periods were identified, including 17 SNPs associated with two indicators, four SNPs with three indicators and four SNPs with 4–5 indicators. The list of these SNPs is presented in Table 5.

### 2.3. Candidate Genes Including PCGs

The identified significant SNPs associated with BW in F_2_ quails of the resource population at different age periods were used to annotate candidate genes associated with the studied parameters (Table 4). In the regions of the identified SNPs (± 0.2 Mb), 419 genes were identified (Supplementary Table S1), including 42 candidate genes in the direct positions of these SNPs. As shown in Table 3, nine PCGs were identified in the positions of 25 SNPs associated with more than one BW index, including *ITM2B* (integral membrane protein 2B), *SLC35F3* (solute carrier family 35 member F3), *ADAM33* (ADAM metallopeptidase domain 33), *UNC79* (unc-79 homolog, NALCN channel complex subunit), *LEPR* (leptin receptor), *RPP14* (ribonuclease P/MRP subunit p14), *MVK* (mevalonate kinase), *ASTN2* (astrotactin 2), and *ZBTB16* (zinc finger and BTB domain containing 16). In 12 PCGs (Table 4), two or more SNPs associated with BW in quails of the studied population were found, including *MARCHF6* (membrane associated ring-CH-type finger 6), *EGFR* (epidermal growth factor receptor), *ADGRL3* (adhesion G protein-coupled receptor L3), *ADAM33*, *NPC2* (NPC intracellular cholesterol transporter 2), *LTBP2* (latent transforming growth factor beta binding protein 2), *ZC2HC1C* (zinc finger C2HC-type containing 1C), *SATB2* (SATB homeobox 2), *ASTN2*, *ZBTB16*, *ADAR* (adenosine deaminase RNA specific), and *LGR6* (leucine rich repeat containing G protein-coupled receptor 6). Moreover, five SNPs were localized within one gene, namely *ADAM33*, for each of which significant associations were established with 3–5 studied indicators, in particular, BW14, BW21, BW28, BW35 and BW42.

PCGs (at *p* < 6.72 × 10^−7^) associated with BW in F_2_ quails of the resource population at different age periods are shown in Table 6.

**Figure 3:**
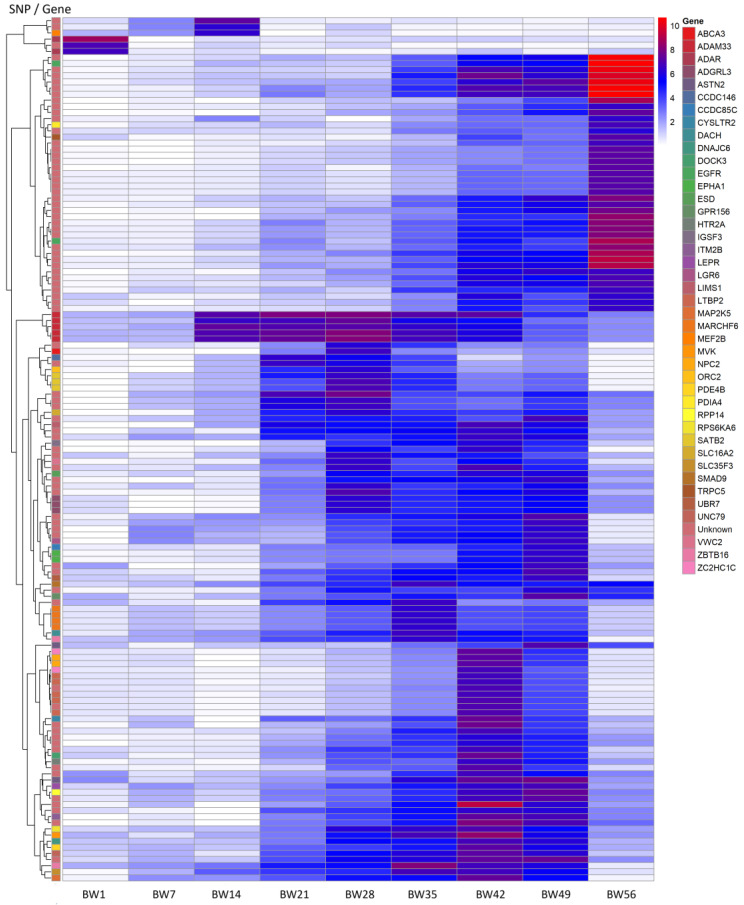
Associations of candidate genes in SNP positions with body weight (BW) of quails at different age periods. *x*-axis: BW indicators at the age of 1, 14, 21, 28, 35, 49 and 56 days; *y*-axis: SNPs and genes in which the SNPs are localized.

Figure 3 represents a summary information on the significant associations of genes established in the positions of the identified SNPs for BW in quails at different age periods.

### 2.4. ADAM33 Association Analysis Example

Taking into account the established significant associations for five SNP loci of the *ADAM33* gene, their allelic variants were examined in more detail, determining the degree of phenotypic manifestation of the BW indicators in quails of the F_2_ resource population in relation to their specific genotypes. The results obtained are presented in Table 7 and Supplementary Table S2.

**Figure 4:**
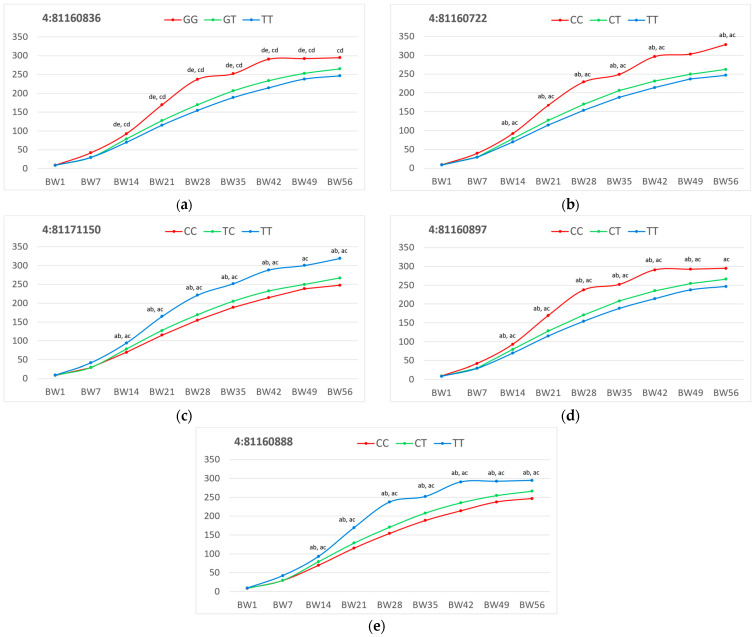
Dynamics of body weight (BW) in F_2_ quails of the resource population depending on the genotype for the *ADAM33* gene at the following loci (chromosome:position): (**a**) 4:81160836, (**b**) 4:81160722, (**c**) 4:81171150, (**d**) 4:81160897 and (**e**) 4:81160888. Along the *x*-axis is BW of quails at the age of 1, 7, 14, 21, 28, 35, 42, 49 and 56 days, and along the *y*-axis is the BW indicator value (in g). BW1, BW7, BW14, BW21, BW28, BW35, BW42, BW49 and BW56 conform to BW in birds aged 1, 7, 14, 21, 28, 35, 42, 49 and 56 days, respectively. Two-letter superscripts (ab, bc, etc.) mean significant differences (at *p* < 0.05) between the following quail groups (for each locus): a, quails with the CC genotype; b, quails with the CT genotype; c, quails with the TT genotype; d, quails with the GG genotype; e, quails with the GT genotype.

BW in quails of the studied population varied depending on the genotype at the loci of the *ADAM33* gene. Significantly higher values of this trait were found in quails with the genotype GG at locus 4:81160836, CC at loci 4:81160722 and 4:81160897, and TT at loci 4:81160888 and 4:81171150 in almost all age periods considered (BW1, BW7, BW14, BW21, BW28, BW35, BW42, BW49 and BW56). Differences in the dynamics of BW of quails of the resource population depending on the genotype are shown in Figure 4.

### 2.5. Gene Ontology Analysis

**Figure 5:**
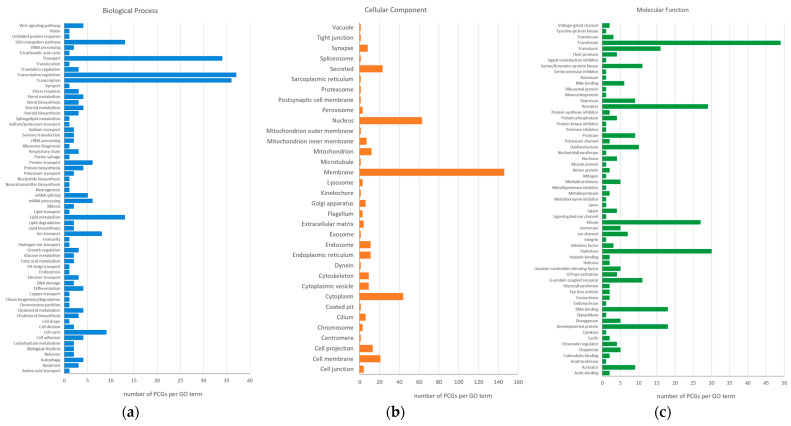
Visualization of the functional annotation of 394 candidate genes for gene ontology (GO) terms according to three main GO aspects: (**a**) biological process, (**b**) cellular component, and (**c**) molecular function. *x*-axis, number of PCGs per GO term.

Of the 419 candidate genes identified in this study, 394 genes, including 42 PCGs, were functionally annotated for gene ontology (GO) terms conforming to three main GO hierarchies, i.e., biological process (GO:BP), cellular component (GO:CC) and molecular function (GO:MF) (Figure 5, Supplementary Figure S2 and Table S3). Accordingly, the most candidate genes were represented in the following GO:BP terms: transcription regulation (37 genes), transcription (36 genes) and transport (34 genes). The GO:CC terms most enriched in candidate genes were membrane (146 genes), nucleus (63 genes) and cytoplasm (44 genes). The most represented GO:MF terms for candidate genes were transferase (49 genes), hydrolase (30 genes), receptor (29 genes) and kinase (27 genes). When only 42 PCGs were considered, most enriched was the GO:CC term membrane (12 genes) (Supplementary Figure S2).

Based on GO term enrichment scores using the DAVID functional annotation tool [77,78], the annotated 394 annotated candidate genes were grouped into 56 functional clusters, six of which had enrichment scores greater than 1.2 (Supplementary Table S3). These clusters included candidate genes related to G-protein coupled receptor, protein tyrosine kinase activator activity, ATP binding, positive regulation of fat cell differentiation, cartilage development, and phosphoprotein. The full list of annotated candidate genes, their functions and clusters are presented in Supplementary Table S3. However, statistically, the GO terms that make up these clusters cannot be accepted as significant and it cannot be considered that the GO analysis has yielded reliable data in our hands. Thus, we have to stipulate that, based on GO term enrichment score analysis, statistical significance was not achieved for them.

## 3. Discussion

Identification of physiological and molecular genetic markers and mechanisms that determine the phenotypic expression of bird body’s potentials pertinent to economically important traits in poultry [79,80,81,82] is essential for modern agriculture. Specifically, it is crucial for the development of genetic methods, efficient genomic selection technologies, and the creation of new highly productive strains and crosses in poultry farming, including quail breeding [83,84,85,86,87,88,89].

BW is an important selected trait for characterizing growth, development and productive qualities of poultry. In meat and egg poultry farming, including quail farming, targeted selection of poultry for this trait has practical significance and demand [35,45,90,91,92,93]. The efficiency of selection of individuals for BW increases significantly when using molecular genetic methods of their assessment by a set of genetic markers that determine the phenotypic manifestation of this trait [52,53,59,71,94,95].

We performed GWAS for the BW indicators in quails at different age periods using a specially created F_2_ resource population. F_2_ resource populations are a convenient model population for molecular genetic studies. Work on the development and use of such model populations is widely carried out in various poultry species, in particular, chickens [21,64,71,73,96], turkeys [17], quails [51,97,98,99], ducks [54]. In our studies, the F_2_ resource population was produced through a series of crosses between two quail breeds that contrasted in the BW index, i.e., the Japanese characterized by a relatively lower growth rate and the Texas White distinguished by a higher rate of BW growth [52,76].

Here, in the course of the conducted GWAS for BW parameters in F_2_ quails of the resource population aged from 1 to 56 days, we established significant associations of the studied characteristics with 142 SNPs and 42 candidate genes localized in the positions of these SNPs. Below we will focus on considering PCGs revealed in our study.

### 3.1. Identification and Relevance of PCGs for BW Parameters

From the viewpoint of using genetic markers in genomic selection, it is of great benefit to search for loci and candidate genes associated with more than one economically, physiologically or adaptively important traits. In this study, SNPs associated with several considered BW traits were localized and the corresponding PCGs in the area of these SNPs were detected. Specifically, 25 SNPs associated with BW in quails at two or more considered ages were determined, including significant associations of four SNPs with BW in 3–5 age periods (BW14, BW21, BW28, BW35, and BW42). In the positions of these 25 SNPs, nine PCGs were identified, including *ITM2B*, *SLC35F3*, *ADAM33*, *UNC79*, *LEPR*, *RPP14*, *MVK*, *ASTN2* and *ZBTB16*. Along with SNPs common to several BW traits considered in this study, we identified 12 PCGs that were co localized with the positions of two and more SNPs associated with the studied traits. These included *MARCHF6*, *EGFR, ADGRL3*, *ADAM33*, *NPC2*, *LTBP2*, *ZC2HC1C*, *SATB2*, *ASTN2*, *ZBTB16*, *ADAR*, and *LGR6*. In all, we established 18 unique PCGs, taking into consideration that *ADAM33*, *ASTN2* and *ZBTB16* were shared between the two groups of PCGs. Below, we consider molecular, physiological and economic relevance of these 18 PCGs in terms of their possible involvement in formation BW-related and other important traits in quails used in our GWAS.

#### 3.1.1. *LEPR* and *ASTN2*

Based on the analysis of open information sources, other studies had previously confirmed the association of the two PCGs, *LEPR* and *ASTN2*, with BW in poultry. For instance, El Moujahid et al. [100] showed an association of the *LEPR* gene with BW in chickens at the age of 49 and 70 days, as well as feed conversion ratio and feed intake. Studies on quails have established the effects of this PCG on the growth indicators of birds at the age of 3 and 5 weeks [101], BW and average daily body weight gain (ADBWG) at the age of 56 days [52]. Li et al. [102] also established the effect of the *LEPR* gene on the deposition of abdominal fat in broilers, the accumulation of which in the body of chickens contributes to an increase in their BW. Among many other examples, SNPs in this PCG were linked to growth and carcass traits in cattle [103,104]. In pigs, *LEPR* effects were demonstrated on body composition [105], productivity and quality traits [106,107], and fatness and growth traits [108]. In sheep, this PCG was associated with age at onset of puberty [109] and reproductive seasonality traits [110]. *ASTN2* has been associated with abdominal circumference in a crossbred commercial pig population [111] and with hair growth and hair follicle development in yaks [112]. Previously, we demonstrated the association of the *ASTN2* gene with BW at 63 days, ADBWG, dressed carcass weight, and drumstick weight in F_2_ resource population chickens [71]. In the present study, we established significant associations of the *LEPR* and *ASTN2* genes with BW in quails of the resource population at the age of 42 and 49 days.

#### 3.1.2. *EGFR* and *ADGRL3*

Two PCGs, *EGFR* and *ADGRL3*, have been shown to be associated with BW and growth in other livestock species. In particular, the *EGFR* gene was linked to weaning weight [113]. The *ADGRL3* gene, which is involved in cell differentiation and energy metabolism, has been associated with growth performance in pigs [114] and sheep [115]. These two PCGs were also found to be involved in the regulation of lipid metabolism. For instance, the *EGFR* gene was related to the regulation of abdominal fat deposition in broilers [116], the proliferation of quail follicular granulosa cells [117] and the regulation of chicken feather follicle morphogenesis [118]. The *ADGRL3* gene was involved in the accumulation of subcutaneous fat in cattle [119]. In the present study, we showed an association of the *ADGRL3* and *EGFR* genes with BW in resource population quails at the age of 28 and 56 days, respectively, which echoes the findings of studies on other animal species.

#### 3.1.3. *MVK*, *NPC2*, *SATB2*, *ADAR*, *ITM2B* and *LTBP2*

A number of studies conducted on farm animals, including poultry, have established an impact of the PCGs identified in this study on the growth, development, and metabolism parameters that determine BW. Specifically, the *MVK* and *NPC2* genes were found to be involved in the regulation of fat deposit accumulation in broiler chickens [120] and pigs [121]. The *SATB2* gene was found to play a role in skeletal muscle development in pigs by regulating myoblast migration [122], gonadal development and ovarian weight in ducks [123] and α- and β-keratin gene cluster switching in chickens [124]. The *ADAR* gene was found to be relevant to bone and muscle development in cattle [125]. Some other PCGs have been shown to be associated with body conformation parameters related to growth and BW. In particular, the *ITM2B* gene was linked to keel length in chickens [69]. In pigs, the *LTBP2* gene was associated with the number of thoracic vertebrae, which determines body length, in F_2_ intercrosses [126] and with teat number [127].

#### 3.1.4. *SLC35F3* and *ADAM33*

Solute carrier SLC35F3 was shown to be one of small molecule transporters that are potentially involved in nutrient absorption and skeletogenesis [128]. ADAM family membrane proteins participate in morphogenesis and tissue formation during chick embryonic development [129,130,131]. *ADAM33* is a gene with a possible function in muscle development and neurogenesis [132], while having also shown preferential expression in smooth muscle, myofibroblasts and fibroblasts of asthmatic airways [132,133]. Davis [134] reported *ADAM33* among 93 candidate genes that exhibited significant differential enrichment in breast muscle of modern Ross 708 broilers relative to the Illinois legacy broiler line. Additionally, it seems to be related to immune system and environmental/climate adaptation in indigenous sheep breeds [132,135].

#### 3.1.5. *ADAR*, *UNC79* and *RPP14*

*ADAR* may play a role in developing body conformation traits such as the rear view of the rear legs in cattle [136]. UNC79 is an auxiliary subunit of the NALCN sodium channel complex (or channelosome) in cell membranes [137,138]; however, its in vivo function in humans is mostly unknown [139]. This PCG may be associated with cold tolerance in chickens [10]. In invertebrate organisms, *UNC79* may play a significant role in the adaptive evolution of mud crabs [140] and regulating starvation resistance in *Drosophila* [141]. *RPP14* may be relevant to muscle growth and functioning as it was up-regulated in *Musculus longissimus dorsi* of steers [142].

#### 3.1.6. *ZBTB16*

The transcription factor ZBTB16 is associated with cell cycle signaling pathways. In particular, it regulates innate and innate-like lymphoid lineage development [143,144,145] and juvenile spermatogonial stem cell development [146]. The *ZBTB16* gene may be related to the regulation of abdominal fat deposition in meat-producing ducks [57]. ZBTB16 also contributes to the bioenergetics of murine brown adipocytes during acute adaptive thermogenesis [147] and showed upregulation in brown adipose tissue and skeletal muscle in cold-stimulated mice, facilitating the deposition of intramuscular fat [148]. Overexpression of *ZBTB16* promotes white adipogenesis and causes the production of brown-like adipocytes in the white intramuscular preadipocytes in *M. longissimus dorsi* of steers [149]. *ZBTB16* was linked to lipid deposition and muscle growth in *M. longissimus dorsi* of pigs according to its demonstrated up-regulated differential expression [150]. Adiposity and BW decreased in rats when one copy of *Zbtb16* was lost [151]. The human body mass index, waist-to-hip ratio and low-density lipoprotein cholesterol levels were all linked to the SNPs in *ZBTB16* [152]. *ZBTB16* might also be a candidate for milk fat yield in dairy cattle [153].

#### 3.1.7. *MARCHF6*, *ZC2HC1C* and *LGR6*

MARCHF6 has been found to be a crucial regulator of a number of metabolic processes, such as protein quality control, cholesterol and lipid droplet homeostasis, and more [154]. The metabolism of lipids and sterols is influenced by its substrates. Post-translationally, MARCHF6 expression is sensitive to the level of cholesterol; elevated cholesterol inhibits its degradation, which raises MARCHF6 levels [155]. *ZC2HC1C* may be related to such important body conformation traits as number of thoracic vertebrae and number of ribs in intercross pig populations [156,157]. It was also identified amongst candidate genes linked to the age at first calving in cattle that are found within the top 1% of genomic windows [158] and may contribute to adaptive traits in the mustelids [159]. In mice, *LGR6* plays a role in skeletal muscles, being transiently expressed during myogenic differentiation and myoblast differentiation and fusion [160]. It also participates in regulating osteogenesis [161].

Collectively, the data obtained in this study pertaining the direct effect of such PCGs as *ADAM33*, *ADGRL3*, *EGFR*, *LEPR* and *ASTN2* on the BW indicators in quails are consistent with the results of other studies conducted both on quails and other species of livestock, including poultry. For some other PCGs identified in our research, a number of previous studies have also shown their relationship with indicators that indirectly characterize and determine BW, e.g., accumulation of fat deposits in the body (*MARCHF6*, *MVK* and *NPC2*), development of muscle and bone tissue (*RPP14*, *SLC35F3*, *SATB2* and *ADAR*), and exterior traits (*ITM2B* and *LTBP2*). Some other PCGs may be beneficial in terms of overall fitness and adaptation in commercially exploited Japanese quails.

The results of this study, conducted at the molecular level, contribute to the expansion of fundamental knowledge about the physiological and molecular genetic mechanisms of formation, development and manifestation of promising performance phenotypes in quails. These serve as a basis for the development of effective technologies for genomic selection, as well as methodological approaches to predicting and assessing the productive potential of quails in order to obtain birds with specified economically important traits. Further studies may be related to the identification of allelic variants of genes in the positions of established SNPs that determine the degree of phenotypic manifestation of BW indicators and growth rate in quails.

## 4. Materials and Methods

### 4.1. Experimental Birds

Hatching eggs of purebred quails were purchased from the Genofond LLC (All-Russian Poultry Research and Technological Institute, Sergiyev Posad, Russia). Birds were hatched and raised at the L. K. Ernst Federal Research Centre for Animal Husbandry (LKEFRCAH) according to generally accepted feeding and maintenance requirements described elsewhere (e.g., [162,163,164]). In particular, the premises used for keeping quails were furnished with the necessary equipment for the full cycle of raising from young to adult birds. The required temperature of 20 to 25 °C and humidity of 55 to 65% were maintained in the premises. At all stages of growing, the birds had free access to compound feed and clean running water. Commercial compound feed was used for feeding in accordance with the age of the birds.

To obtain the F_2_ resource population, a series of crosses were carried out using two breeds with contrasting BW phenotype and growth rate, i.e., Japanese (of lower BW and growth rate) and Texas White (of higher BW and growth rate). In more detail, the F_2_ resource population was based on the original parental breeds characterized by a higher growth rate (Texas White) and a slower growth (Japanese). At all stages of producing the F_2_ resource population, strict accounting of kinship was carried out to exclude inbreeding. Initially, four families (F0_1–F0_4) were formed, each of which included one male and five females. In groups F0_1 and F0_2, Japanese males were used as a paternal parental stock, and Texas White females as a maternal stock. In groups F0_3 and F0_4, the reciprocal combination of parental breeds was used, i.e., Texas White males mated to Japanese females. From F0 individuals, F1 quails were obtained (F1_1–F1_4) that were used to deliver the F_2_ resource population. For this purpose, eight F1 families were formed in the following combinations: groups 1 and 2, one male F1_1 and three females F1_2; groups 3 and 4, one male F1_2 and three females F1_1; groups 5 and 6, one male F1_3 and three females F1_4; and groups 7 and 8, one male F1_4 and three females F1_3. A total of 640 F_2_ quails were produced. To search for significant associations of SNPs and candidate genes with BW, a model resource population was formed within the framework of this study, including 240 F_2_ female quails. When forming this experimental sampling, contrasting phenotypes of F_2_ female quails by BW at the age of 8 weeks were taken into account. To characterize the genetic structure of this population, F_2_ individuals were divided into eight groups (F2_1 to F2_8) depending on the F_1_ males used to produce them, and their DNA samples were collected.

### 4.2. Phenotypic Traits and Their Statistical Analyses

F_2_ quails from the resource population (*n* = 240) were phenotyped for BW1, BW7, BW14, BW28, BW35, BW42, BW49 and BW56. Weighing was performed by qualified personnel using electronic scales with a measurement error of 0.01 g. To assess the growth dynamics of the F_2_ resource population in comparison with the original parental breeds, BW measurement was conducted using Japanese (*n* = 30) and Texas White (*n* = 20) quails at the same ages of 1, 7, 14, 21, 28, 35, 42 and 49 days.

To calculate the heritability indices for BW of individuals for the age periods from Day 1 to Day 56 and genetic correlations between them, a mixed model equation was developed using the BLUP Animal Model methodology based on the calculation of variance-covariance complexes using the REML (restricted maximum likelihood) procedure. A multi-trait model was used in the analysis. These calculations were performed using the BLUPF90 family of programs [165].

The mixed model equation was utilized as follows:

(1) $$y_{ijkl}=\;\mu +{PG}_{i}+{Hatch}_{j}+{Sex}_{k}+{animal}_{l}+e_{ijkl},$$

where *y* is the value of the resultant BW trait (from 1 to 56 days with a step of 7 days) for individual *l* in the *i*-th group from the parent family of the resource population, the *j*-th hatching batch of during incubation and the *k*-th sex; *µ* is the average value of the trait for each age group from the studied sample of the quail resource population; *PG* is the fixed effect of the *i*-th group from the parent family of the resource population; *Hatch* is the fixed effect of the *j*-th hatching batch of chicks during incubation; *Sex* is the fixed effect of the *k*-th sex of an individual; *animal* is the randomized (random) effect of individual *l* from the F_2_ resource population; and *e* is the residual (undistributed) variance of the equation model.

The heritability coefficient (*h*^2^) was calculated using the following formula:

(2) $$h^{2}=\frac{VarA}{VarA+VarE},$$

where *VarA* is the genetic variance of the model equation for the studied trait; and *VarE’* is the residual variance of the model equation for the studied trait.

### 4.3. Sampling and DNA Extraction

DNA was extracted from the feather pulp of F_2_ quails of the resource population using the Syntol kit for animal tissue (Syntol, Moscow, Russia). The concentration of the isolated DNA was assessed using a Qubit 3.0 fluorimeter (Thermo Fisher Scientific, Wilmington, DE, USA), and the purity of the extracted DNA (based on the OD260/280 ratio) was assessed using a Thermo Fisher Scientific’s NanoDrop-2000 spectrophotometer.

### 4.4. Sequencing, Genotyping and SNP Quality Control

Genotyping of quails was carried out using the GBS technique according to the protocol by Elshire et al. [166], with some modifications proposed by Dodds et al. [167]. In the course of the study, a GBS library was prepared using double digestion with *Pst*I–*Msp*I restriction enzymes, including negative controls without DNA. The Pippin Prep system (SAGE Science, Beverly, MA, USA) was used to select DNA fragments of 220–340 bp, taking into account the length of the genomic sequence along with adapters (148 bp). A set of 768 unique adapter sequences (manufactured by Illumina, San Diego, CA, USA and Integrated DNA Technologies Coralville, IA, USA) were used and contained 10-nucleotide barcodes differing by at least three mutational steps. The corresponding adapter sequences were used as follows: PstI_barcode_adapter_F, ACACTCTTTCCCTACACGACGCTCTTCCGATCTNNNNNNNNNNTGCA; PstI_barcode_adapter_R, NNNNNNNNNNAGATCGGAAGAGCGTCGTGTAGGGAAAGAGTGT; MspI(Y)_Common_F, CGAGATCGGAAGAGCGGACTTTAAGC; and MspI_Common_R, GTGACTGGAGTTCAGACGTGTGCTCTTCCGATCT. Sequencing was performed on the Illumina NovaSeq 6000 platform using v1.5 reagents in the single-read mode (101 nucleotides). Quality control of the generated data was performed using the DECONVQC algorithm [168,169] and FastQC software [170]. The mean GBS coverage depth was 0.269.

The Japanese quail Coturnix japonica 2.0 (CJA 2.0) genome assembly [171] from the Ensembl 104 and Ensembl Genomes 51 databases [172] was used as a reference genome. Data processing was performed using cutadapt to remove adapter sequences and demultiplex by barcodes [173,174]. The quality of fastq files was additionally checked using FastQC [170]. Read alignment to the reference genome was executed using bowtie2 (version 2.4.4; [175]), and subsequent processing and sorting of BAM files was performed using samtools [176].

Joint genotyping of the output files was performed using commands from the snpGBS [177] and bamtools [176] packages to generate a single VCF file (bcftools mpileup … | bcftools call-m—| bcftools view-M2 …). Using R software (version 4.0.0; [178,179]), the data were presented in a format suitable for further analysis. PLINK 1.9 [180,181] was used to quality control the SNP detection. Quail genotypes were filtered according to the genotyping efficiency parameter (MAF 0.03), and SNPs that were genotyped in fewer than 90% of samples (genotype 0.1) were not included in the study.

Quality control and filtering of the GBS genotyping data for each sample and each SNP were performed in the R software environment (version 4.0.0; [178,179]) using the PLINK 1.9 software package [180,181], applying the following filters in the program: --mind 0.10, --geno 0.10, --maf 0.03, --hwe 1e-6. After filtering, 74,387 SNPs were used for further analysis.

### 4.5. PCA Procedure

PCA [182] was performed in PLINK 1.9. Data files were prepared in the R software environment (version 4.0.0; [178,179]). Visualization of the obtained results by plotting the respective graphs was carried out in the R package (version 3.5.1) ggplot2 [183,184].

### 4.6. Genome-Wide Association Study and GO Analysis

The search for SNP associations with BW in F_2_ quails of the resource population was carried out using regression analysis in PLINK 1.9. The significance of the SNP effects and the identification of significant regions in the quail genome were assessed using the Bonferroni null hypothesis test at a threshold of *p* < 6.72 × 10^−7^. Data were visualized in the qqman package (version 0.1.9) [185] using the R programming language [186].

In the position and region (±0.2 Mb) of the identified SNPs, a search for candidate genes was performed using the CJA 2.0 genome assembly resource [171,172].

The Ensembl BioMart data mining tool and the Database for Annotation, Visualization, and Integrated Discovery (DAVID Knowledgebase; version DAVID 2021 (December 2021), v2023q4, updated quarterly) [77,78] were used to conduct functional annotation and GO term enrichment analyses for the whole candidate gene set and for the prime candidate genes (PCGs). Functional annotation visualization of candidate genes for GO terms relative to three main GO aspects, i.e., biological process, cellular component and molecular function, was performed using Microsoft Excel.

## 5. Conclusions

In this study, we conducted a GWAS for the BW traits in F_2_ Japanese quails of the resource population based on GBS genotyping data and identified relevant molecular markers and candidate genes. In the course of the conducted study, 142 significant SNPs and, correspondingly, 42 candidate genes were found to have a high significant association with BW in quails at the age of 1 (three SNPs, one gene), 14 (eight SNPs, two genes), 21 (eight SNPs, two genes), 28 (23 SNPs, seven genes), 35 (15 SNPs, seven genes), 42 (50 SNPs, 22 genes), 49 (31 SNPs, 11 genes) and 56 (42 SNPs, three genes) days. A significant proportion of the determined SNPs and candidate genes were detected on chromosomes CJA1, CJA4 and CJA5. We established 25 SNPs and nine related PCGs that were associated with BW in two or more age periods, including four SNPs associated with 4–5 BW indices. For 12 PCGs, i.e., *MARCHF6*, *EGFR*, *ADGRL3*, *ADAM33*, *NPC2*, *LTBP2*, *ZC2HC1C*, *SATB2*, *ASTN2*, *ZBTB16*, *ADAR* and LGR6, two or more SNPs associated with the studied indices were detected. Herewith, there was one gene, *ADAM33*, for which five SNPs were found that had significant associations with 3–5 indices (BW14, BW21, BW28, BW35, and BW42). Our findings are of great importance for understanding the genetic basis for the formation and manifestation of growth potential in quails. The identified significant SNPs and putative candidate genes require further study and can be used as molecular genetic markers in breeding programs aimed at increasing and improving growth performance and productivity in Japanese quail.

## Figures and Tables

**Table 1:** Descriptive statistics for body weight (BW) indices (in g) in quails of the F_2_ resource population and the original parent breeds.

Traits ^1^	F_2_ (*n* = 240)			Japanese Breed (*n* = 30)			Texas White Breed (*n* = 20)		
	Mean	SD ^2^	CV ^3^, %	Mean	SD	CV, %	Mean	SD	CV, %
BW1	8.8	0.9	10.5	8.5	0.4	4.2	9.3	0.8	8.6
BW7	31.7	6.3	21.7	28.9	3.2	10.1	40.0	3.7	9.4
BW14	77.4	12.1	17.4	69.3	7.4	9.6	93.2	4.2	4.5
BW21	116.4	19.6	16.8	103.8	18.4	17.7	157.8	25.7	16.3
BW28	158.5	22.1	13.9	125.5	24.8	19.8	204.9	32.1	15.6
BW35	191.7	25.2	13.2	164.5	22.4	13.6	255.7	29.3	11.5
BW42	218.9	29.6	13.5	174.5	18.1	10.4	275.6	37.3	13.5
BW49	241.4	30.1	12.5	205.8	32.0	15.5	301.0	41.2	13.7
BW56	249.3	33.2	13.3	–	–	–	–	–	–

^1^ BW1, BW7, BW14, BW21, BW28, BW35, BW42, BW49 and BW56 conform to BW in birds aged 1, 7, 14, 21, 28, 35, 42, 49 and 56 days, respectively. ^2^ SD, standard deviation. ^3^ CV, coefficient of variation.

**Table 2:** Heritability, genetic and residual variance indices for body weight of the F_2_ resource population quails of different ages.

Traits ^1^	Genetic Variance (*VarA*)	Residual Variance (*VarE*)	Heritability (*h*^2^)
BW1	0.395	0.304	0.565
BW7	13.49	24.69	0.353
BW14	48.22	106.2	0.312
BW21	87.44	226.4	0.279
BW28	122.9	310.4	0.284
BW35	207.8	500.4	0.293
BW42	234.9	577.3	0.289
BW49	246.2	627.3	0.282
BW56	281.0	732.0	0.277

^1^ BW1, BW7, BW14, BW21, BW28, BW35, BW42, BW49 and BW56 conform to BW in birds aged 1, 7, 14, 21, 28, 35, 42, 49 and 56 days, respectively.

**Table 3:** Genetic correlations between body weight (BW) indicators in quails of the F_2_ resource population.

Traits ^1^	BW1	BW7	BW14	BW21	BW28	BW35	BW42	BW49	BW56
BW1	–								
BW7	0.039	–							
BW14	0.074	0.518	–						
BW21	0.049	0.427	0.680	–					
BW28	0.026	0.386	0.659	0.665	–				
BW35	0.026	0.336	0.570	0.628	0.702	–			
BW42	0.008	0.314	0.542	0.543	0.668	0.711	–		
BW49	−0.002	0.314	0.487	0.494	0.580	0.629	0.744	–	
BW56	0.009	0.308	0.428	0.436	0.492	0.508	0.601	0.751	–

^1^ BW1, BW7, BW14, BW21, BW28, BW35, BW42, BW49 and BW56 conform to BW in birds aged 1, 7, 14, 21, 28, 35, 42, 49 and 56 days, respectively.

**Table 4:** Distribution of significant single nucleotide polymorphisms (SNPs; at *p* < 6.72 × 10^−7^) associated with body weight (BW) on chromosomes in F_2_ quails of the resource population.

Traits ^1^	No. of SNPs	Chromosomes
BW1	3	CJA5, CJA25
BW14	8	CJA4, CJA7, CJA26, CJA28
BW21	8	CJA4, CJA7, CJA10
BW28	23	CJA2, CJA4, CJA7, CJA8, CJA10, CJA11, CJA14, CJA15, CJA22
BW35	15	CJA1, CJA2, CJA3, CJA4, CJA15, CJA24
BW42	50	CJA1, CJA3-CJA6, CJA8, CJA10, CJA12, CJA15, CJA17, CJA18, CJA20, CJA22, CJA24, CJA26
BW49	31	CJA1, CJA5, CJA8, CJA12, CJA17, CJA20, CJA22, CJA23, CJA26
BW56	42	CJA1-CJA4, CJA8, CJA14, CJA15, CJA18, CJA20, CJA25, CJA26

^1^ BW1, BW7, BW14, BW21, BW28, BW35, BW42, BW49 and BW56 conform to BW in birds aged 1, 7, 14, 21, 28, 35, 42, 49 and 56 days, respectively.

**Table 5:** List of significant single nucleotide polymorphisms (SNPs; at *p* < 6.72 × 10^−7^) associated with body weight (BW) indices in F_2_ quails of the resource population in two or more age periods and prioritized candidate genes (PCGs).

Chromosome	SNP Position (in bp)	Traits ^1^	PCG (at SNP Position)
CJA1	151,007,527	BW42, BW49	*ITM2B*
CJA1	151,084,995	BW42, BW49	–
CJA3	34,595,032	BW35, BW42	*SLC35F3*
CJA4	81,160,722	BW14, BW21, BW28, BW35, BW42	*ADAM33*
CJA4	81,160,836	BW14, BW21, BW28	*ADAM33*
CJA4	81,160,888	BW14, BW21, BW28, BW35	*ADAM33*
CJA4	81,160,897	BW14, BW21, BW28, BW35	*ADAM33*
CJA4	81,171,150	BW14, BW21, BW28, BW35	*ADAM33*
CJA5	40,981,270	BW42, BW49	*UNC79*
CJA8	24,869,812	BW28, BW42	–
CJA8	25,524,051	BW42, BW49	*LEPR*
CJA8	25,548,415	BW42, BW49	–
CJA10	10,505,840	BW21, BW28	–
CJA12	9,300,025	BW42, BW49	*RPP14*
CJA12	9,302,400	BW42, BW49	–
CJA15	5,904,595	BW35, BW42	*MVK*
CJA15	11,552,880	BW42, BW56	–
CJA15	11,552,881	BW42, BW49, BW56	–
CJA15	11,622,693	BW49, BW56	–
CJA17	2,261,513	BW42, BW49	*ASTN2*
CJA20	4,345,582	BW49, BW56	–
CJA24	3,829,762	BW35, BW42	*ZBTB16*
CJA26	68,898	BW49, BW56	–
CJA26	243,899	BW42, BW49, BW56	–
CJA26	243,912	BW42, BW49, BW56	–

^1^ BW1, BW7, BW14, BW21, BW28, BW35, BW42, BW49 and BW56 conform to BW in birds aged 1, 7, 14, 21, 28, 35, 42, 49 and 56 days, respectively.

**Table 6:** Prioritized candidate genes (PCGs) colocalized with more than one single nucleotide polymorphism (SNP) and associated with body weight (BW) in F_2_ quails of the resource population.

Chromosome	PCG	SNP Position (in bp)	Traits ^1^	*p*-Value
CJA2	*MARCHF6*	52,281,470	BW35	5.35 × 10^−7^
		52,281,495	BW35	5.35 × 10^−7^
		52,281,496	BW35	5.35 × 10^−7^
		52,281,497	BW35	5.35 × 10^−7^
	*EGFR*	73,896,881	BW56	2.12 × 10^−11^
		73,896,952	BW56	1.32 × 10^−9^
CJA4	*ADGRL3*	43,060,269	BW28	6.18 × 10^−7^
		43,060,333	BW28	6.18 × 10^−7^
		43,060,345	BW28	6.18 × 10^−7^
	*ADAM33*	81,160,722	BW14	9.46 × 10^−8^
			BW21	1.59 × 10^−8^
			BW28	1.36 × 10^−8^
			BW35	8.27 × 10^−8^
			BW42	7.96 × 10^−8^
		81,160,836	BW14	4.46 × 10^−8^
			BW21	3.39 × 10^−7^
			BW28	4.27 × 10^−8^
		81,160,888	BW14	1.20 × 10^−7^
			BW21	5.73 × 10^−8^
			BW28	9.55 × 10^−9^
			BW35	2.64 × 10^−7^
		81,160,897	BW14	1.20 × 10^−7^
			BW21	5.73 × 10^−8^
			BW28	9.55 × 10^−9^
			BW35	2.64 × 10^−7^
		81,171,150	BW14	6.12 × 10^−8^
			BW21	1.35 × 10^−7^
			BW28	8.41 × 10^−8^
			BW35	4.58 × 10^−7^
CJA5	*NPC2*	34,403,827	BW42	8.89 × 10^−8^
		34,403,875	BW42	8.89 × 10^−8^
	*LTBP2*	34,409,135	BW42	1.70 × 10^−7^
		34,409,184	BW42	3.45 × 10^−7^
		34,411,616	BW42	1.70 × 10^−7^
		34,412,981	BW42	3.07 × 10^−7^
		34,417,379	BW42	2.75 × 10^−7^
	*ZC2HC1C*	34,609,626	BW42	2.72 × 10^−7^
		34,610,313	BW42	6.96 × 10^−8^
CJA7	*SATB2*	9,470,112	BW28	3.91 × 10^−7^
		9,470,188	BW28	1.30 × 10^−7^
		9,470,245	BW28	1.30 × 10^−7^
CJA17	*ASTN2*	2,218,419	BW49	5.83 × 10^−8^
		2,261,513	BW42	6.15 × 10^−8^
			BW49	2.61 × 10^−8^
CJA24	*ZBTB16*	3,829,762	BW35	1.05 × 10^−8^
			BW42	2.36 × 10^−7^
		3,847,375	BW35	5.43 × 10^−7^
CJA25	*ADAR*	1,503,153	BW1	2.82 × 10^−7^
		1,503,237	BW1	3.14 × 10^−9^
CJA26	*LGR6*	798,927	BW49	3.76 × 10^−7^
		850,193	BW49	2.94 × 10^−7^

^1^ BW1, BW7, BW14, BW21, BW28, BW35, BW42, BW49 and BW56 conform to BW in birds aged 1, 7, 14, 21, 28, 35, 42, 49 and 56 days, respectively.

**Table 7:** Body weight (BW) values (mean ± standard error of the mean) in F_2_ quails of the resource population depending on the genotype at five single nucleotide polymorphism (SNP) loci (chromosome:position) of the *ADAM33* gene.

SNP Geno-type	*n* ^1^	Traits ^2^								
		BW1	BW7	BW14	BW21	BW28	BW35	BW42	BW49	BW56
4:81160722										
CC	13	9.2 ± 0.2	39.7 ± 1.2	91.9 ± 1.0 ^ab, ac^	167.0 ± 3.8 ^ ab, ac^	228.8 ± 5.5 ^ ab, ac^	249.0 ± 5.2 ^ ab, ac^	296.2 ± 9.7 ^ ab, ac^	302.8 ± 9.7	328.0 ± 8.2 ^ ab, ac^
CT	47	8.8 ± 0.1	29.4 ± 1.0	78.5 ± 1.8 ^ab, bc^	127.3 ± 3.7 ^ bc^	169.7 ± 4.1 ^ ab, bc^	206.4 ± 4.4 ^ ab, bc^	231.5 ± 4.9 ^ ab, bc^	249.4 ± 5.4	262.4 ± 5.7 ^ab^
TT	173	8.5 ± 0.1	29.1 ± 0.5	69.5 ± 0.9 ^ ac^	114.5 ± 1.5 ^ ac^	153.6 ± 1.9 ^ ac^	188.0 ± 2.1 ^ ac^	214.1 ± 2.4 ^ ac^	236.8 ± 2.2	246.8 ± 2.4 ^ ac^
4:81160836										
GG	11	9.0 ± 0.2	42.0 ± 1.4	92.7 ± 1.4 ^de, cd^	169.3 ± 5.5 ^ de, cd^	237.3 ± 5.9 ^ de, cd^	252.0 ± 5.8 ^ de, cd^	290.7 ± 10.4 ^ de, cd^	292.3 ± 10.9 ^ de, cd^	294.8 ± 8.9 ^ cd^
GT	50	8.7 ± 0.1	29.4 ± 1.0	78.7 ± 1.8 ^ce, de^	127.3 ± 3.8 ^ce, de^	169.5 ± 4.0 ^ ce, de^	206.4 ± 4.3 ^ ce, de^	233.6 ± 5.0 ^ ce, de^	253.1 ± 5.5 ^ ce^	265.1 ± 5.8 ^ ce, de^
TT	172	8.5 ± 0.1	29.3 ± 0.5	69.5 ± 0.9 ^cd, ce^	115.1 ± 1.5 ^ cd, ce^	154.4 ± 1.9 ^ cd, ce^	188.6 ± 2.2 ^ cd, ce^	214.6 ± 2.4 ^ cd, ce^	237.9 ± 2.5 ^ cd^	246.4 ± 2.3 ^ cd, ce^
4:81160888										
CC	171	8.5 ± 0.1	29.2 ± 1.4	69.4 ± 0.9 ^ab, ac^	114.9 ± 1.5 ^ ab, ac^	154.1 ± 1.9 ^ ab, ac^	188.3 ± 2.1 ^ ab, ac^	214.2 ± 2.4 ^ ab, ac^	237.6 ± 2.5 ^ ab, ac^	246.7 ± 2.5 ^ ab, ac^
TC	51	8.8 ± 0.1	29.7 ± 1.0	79.1 ± 1.8 ^ab, bc^	128.5 ± 3.8 ^ ab, bc^	170.7 ± 4.0 ^ ab, bc^	208.1 ± 4.4 ^ ab, bc^	235.3 ± 5.1 ^ ab, bc^	254.4 ± 5.5 ^ ab, bc^	266.1 ± 5.4 ^ ab^
TT	11	9.0 ± 0.2	42.0 ± 0.2	92.7 ± 1.4 ^ac, bc^	169.3 ± 5.5 ^ ac, bc^	237.3 ± 5.9 ^ ac, bc^	252.0 ± 5.8 ^ ac, bc^	290.7 ± 10.4 ^ ac, bc^	292.3 ± 10.9 ^ ac, bc^	294.8 ± 8.9 ^ ac^
4:81160897										
CC	11	9.0 ± 0.2	42.0 ± 1.4	92.7 ± 1.4 ^ab, ac^	169.3 ± 5.5 ^ ab, ac^	237.3 ± 5.9 ^ ab, ac^	252.0 ± 5.8 ^ ab, ac^	290.7 ± 10.4 ^ ab, ac^	292.3 ± 10.9 ^ ab, ac^	294.8 ± 8.9 ^ ac^
CT	51	8.8 ± 0.1	29.7 ± 1.0	79.1 ± 1.8 ^ab, bc^	128.5 ± 3.8 ^ ab, bc^	170.7 ± 4.0 ^ ab, bc^	208.1 ± 4.4 ^ ab, bc^	235.3 ± 5.1 ^ ab, bc^	254.4 ± 5.5 ^ ab, bc^	266.1 ± 5.4 ^ bc^
TT	171	8.5 ± 0.1	29.2 ± 0.5	69.4 ± 0.9 ^ac, bc^	114.9 ± 1.5 ^ ac, bc^	154.1 ± 1.9 ^ ac, bc^	188.3 ± 2.1 ^ ac, bc^	214.2 ± 2.4 ^ ac, bc^	237.6 ± 2.5 ^ ac, bc^	246.7 ± 2.5 ^ ac, bc^
4:81171150										
CC	174	8.5 ± 0.1 ^ac^	29.2 ± 0.5	69.5 ± 0.9 ^ab, ac^	115.2 ± 1.5 ^ ab, ac^	154.8 ± 1.9 ^ ab, ac^	189.0 ± 2.1 ^ ab, ac^	214.8 ± 2.4 ^ ab, ac^	238.4 ± 2.5 ^ac^	247.6 ± 2.5 ^ ab, ac^
TC	45	8.7 ± 0.1	29.0 ± 1.0	77.9 ± 1.8 ^ab, bc^	127.4 ± 4.0 ^ ab, bc^	169.1 ± 4.4 ^ ab, bc^	205.0 ± ±4.4 ^ ab, bc^	232.1 ± 5.3 ^ ab, bc^	249.7 ± 5.5 ^ bc^	267.0 ± 5.9 ^ ab, bc^
TT	14	9.3 ± 0.2 ^ac^	41.8 ± 1.2	94.3 ± 1.4 ^ac, bc^	164.8 ± 4.6 ^ ac, bc^	221.5 ± 6.2 ^ ac, bc^	251.8 ± 5.5 ^ ac, bc^	288.3 ± 10.8 ^ ac, bc^	300.3 ± 10.0 ^ ac, bc^	319.1 ± 8.7 ^ ac, bc^

^1^ *n*, number of birds; ^2^ BW1, BW7, BW14, BW21, BW28, BW35, BW42, BW49 and BW56 conform to BW in birds aged 1, 7, 14, 21, 28, 35, 42, 49 and 56 days, respectively. Two-letter superscripts (^ab^, ^bc^, etc.) mean significant differences (at *p* < 0.05) between the following quail groups (for each locus): a, quails with the CC genotype; b, quails with the CT genotype; c, quails with the TT genotype; d, quails with the GG genotype; e, quails with the GT genotype.

## Data Availability

The original contributions presented in this study are included in the article/Supplementary Material. Further inquiries can be directed to the corresponding author(s). The sequence data are accessible to readers on request.

## Supplementary Materials

The following supporting information can be downloaded at: https://www.mdpi.com/article/10.3390/ijms26178243/s1.

## Abbreviations

The following abbreviations are used in this manuscript:

BW	body weight
SNP(s)	single nucleotide polymorphism(s)
GWAS	genome-wide association study
PCG(s)	prioritized candidate gene(s)
GBS	genotyping-by-sequencing
CJA	*Coturnix japonica*
BW1, BW7, BW14, BW21, BW28, BW35, BW42, BW49, BW56	1, 7, 14, 21, 28, 35, 42, 49 and 56 days of age
SD	standard deviation
CV	coefficient of variation
PCA	principal component analysis
PC1	first component
PC2	second component
PC3	third component
*ITM2B*	integral membrane protein 2B
*SLC35F3*	solute carrier family 35 member F3
*ADAM33*	ADAM metallopeptidase domain 33
*UNC79*	unc-79 homolog, NALCN channel complex subunit
*LEPR*	leptin receptor
*RPP14*	ribonuclease P/MRP subunit p14
*MVK*	mevalonate kinase
*ASTN2*	astrotactin 2
*ZBTB16*	zinc finger and BTB domain containing 16
*MARCHF6*	membrane associated ring-CH-type finger 6
*EGFR*	epidermal growth factor receptor
*ADGRL3*	adhesion G protein-coupled receptor L3
*NPC2*	NPC intracellular cholesterol transporter 2
*LTBP2*	latent transforming growth factor beta binding protein 2
*ZC2HC1C*	zinc finger C2HC-type containing 1C
*SATB2*	SATB homeobox 2
*ADAR*	adenosine deaminase RNA specific
*LGR6*	leucine rich repeat containing G protein-coupled receptor 6
GO	gene ontology
ADBWG	average daily body weight gain
LKEFRCAH	L. K. Ernst Federal Research Centre for Animal Husbandry
