# Supplementary material for: Genome-Wide Association Studies in Japanese Quails of the F2 Resource Population Elucidate Molecular Markers and Candidate Genes for Body Weight Parameters
Source: Int J Mol Sci. 2025 Aug 25;26(17):8243. doi: 10.3390/ijms26178243 (PMC12427666; doi:10.3390/ijms26178243)
Supplement: Supplementary file 1 [file ijms-26-08243-s001.zip › Supplementary Figure S1.pdf]

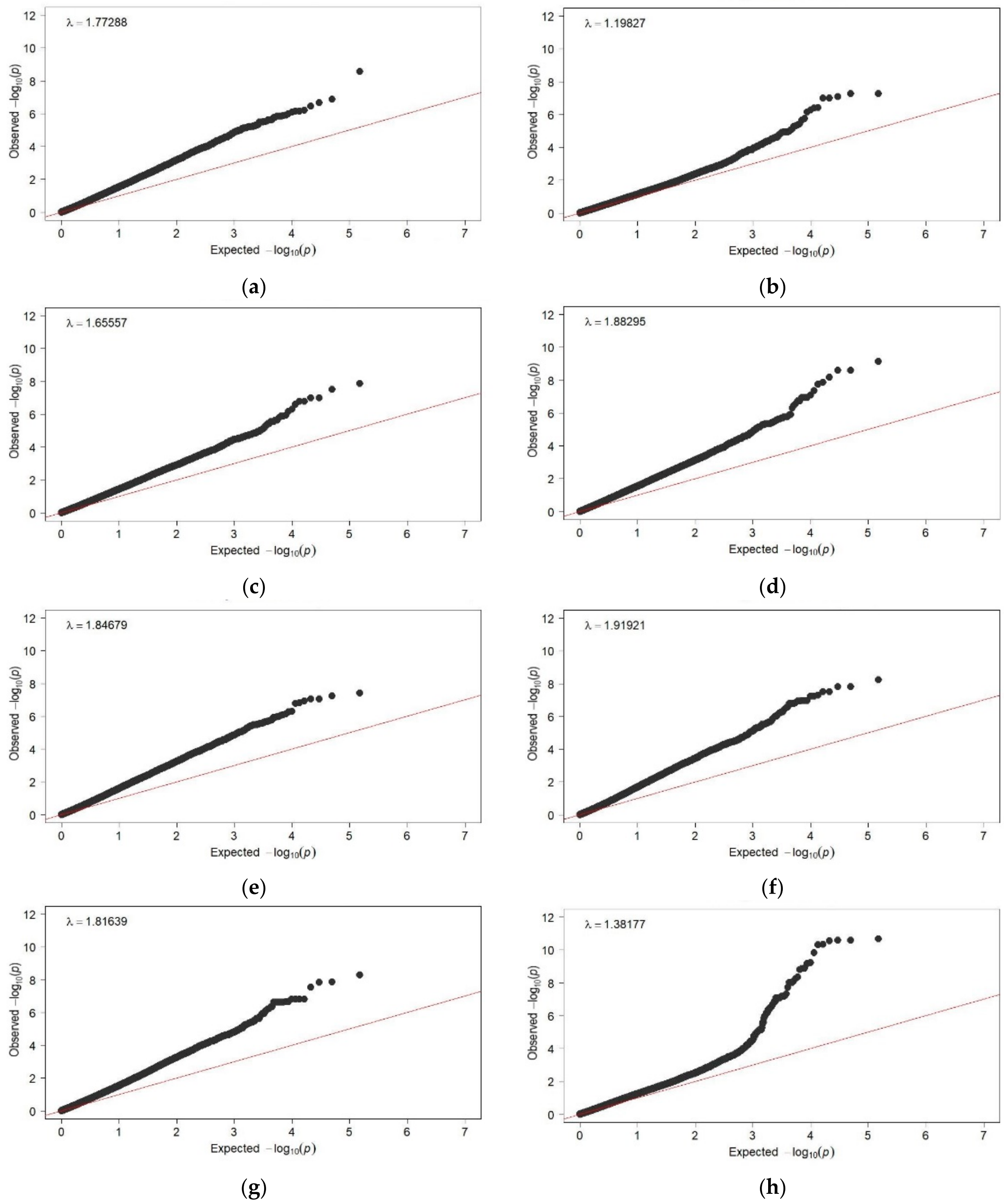

Supplementary Figure S1. Quantile–quantile (Q–Q) plots and the respective λ values for the GWAS results based on the studied body weight (BW) parameters in the F_2 resource population of quails at the ages of 1 (**a**), 14 (**b**), 21 (**c**), 28 (**d**), 35 (**e**), 42 (**f**), 49 (**g**) and 56 (**h**) days. Q–Q plots represented quantiles of the probability distribution of expected and observed deviations from the normal distribution for nominal significance values ($-\log_{10}(p)$).
