# Supplementary material for: Genome-Wide Association Studies in Japanese Quails of the F2 Resource Population Elucidate Molecular Markers and Candidate Genes for Body Weight Parameters
Source: Int J Mol Sci. 2025 Aug 25;26(17):8243. doi: 10.3390/ijms26178243 (PMC12427666; doi:10.3390/ijms26178243)
Supplement: Supplementary file 1 [file ijms-26-08243-s001.zip › Supplementary Figure S2.pdf]

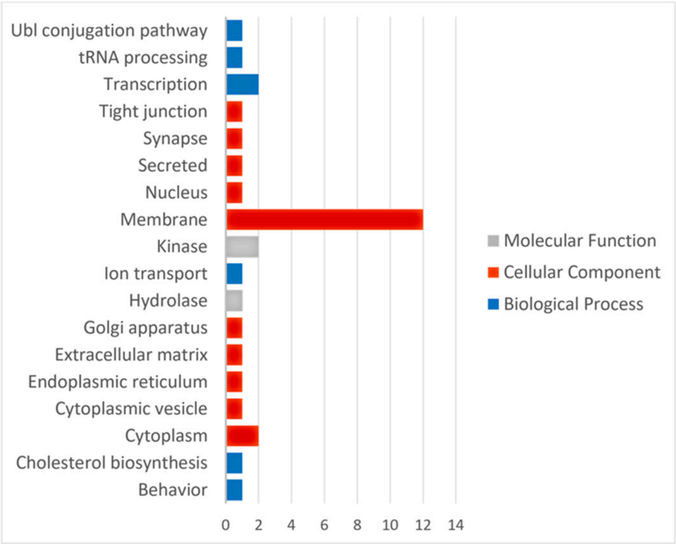

Supplementary Figure S2. Functional annotation visualization of 42 prioritized candidate genes (PCGs) for gene ontology (GO) terms relative to three main GO aspects, i.e., biological process, cellular component, and molecular function. *x*-axis, number of PCGs per GO term.
